# B7H3 Immune Checkpoint Overexpression Is Associated with Decreased Complete Response Rates to Neoadjuvant Therapy in Locally Advanced Rectal Cancer

**DOI:** 10.3390/diagnostics14182023

**Published:** 2024-09-12

**Authors:** Sebastian Curcean, Raluca Maria Hendea, Rares Buiga, Alexandru Tipcu, Andra Curcean, Catalin Vlad, Zsolt Fekete, Alina-Simona Muntean, Daniela Martin, Alexandru Irimie

**Affiliations:** 1 Department of Radiation Oncology, Iuliu Hatieganu University of Medicine and Pharmacy, 400012 Cluj-Napoca, Romania; 2 Department of Radiation Oncology, “Prof. Dr. Ion Chiricuta” Oncology Institute, 400015 Cluj-Napoca, Romania; 3 Department of Pathology, Iuliu Hatieganu University of Medicine and Pharmacy, 400012 Cluj-Napoca, Romania; 4 Department of Pathology, “Prof. Dr. Ion Chiricuta” Oncology Institute, 400015 Cluj-Napoca, Romania; 5 Faculty of Medicine, Iuliu Hatieganu University of Medicine and Pharmacy, 400012 Cluj-Napoca, Romania; 6 Department of Imaging, Affidea Center, 400487 Cluj-Napoca, Romania; 7 Department of Oncological Surgery and Gynecological Oncology, Iuliu Hatieganu University of Medicine and Pharmacy, 400012 Cluj-Napoca, Romania; 8 Department of Oncological Surgery, “Prof. Dr. Ion Chiricuta” Oncology Institute, 400015 Cluj-Napoca, Romania

**Keywords:** B7H3, locally advanced rectal cancer, radiotherapy, complete response

## Abstract

Background and Objectives: Rectal cancer accounts for approximately one-third of colorectal cancers, with over 340,000 deaths globally in 2022. Despite advancements in treatment, the five-year overall survival for locally advanced rectal cancer (LARC) remains at 74%, with significant morbidity. B7H3 (CD276), an immune checkpoint protein, plays a role in tumor progression and resistance to therapy, and correlates with poor prognosis in various cancers, including colorectal cancer. This study aims to evaluate the expression of B7H3 in LARC and its impact on overall complete response (oCR) rates to neoadjuvant therapy. Methods: A retrospective study was conducted on 60 patients with LARC who received neoadjuvant chemoradiation (nCRT) followed by total mesorectal excision (TME). B7H3 expression was assessed using immunohistochemistry on surgical specimens. Expression levels were categorized as high or low based on a composite score, and their association with oCR rates was analyzed. Results: High B7H3 expression was observed in 60% of patients, with 73.5% showing expression in more than 50% of tumor cells. Patients who achieved oCR had significantly lower B7H3 expression compared to those with residual disease (*p* < 0.001). No nuclear expression of B7H3 was detected. No significant correlation was found between B7H3 expression and other clinicopathological variables, except for a higher likelihood of non-restorative surgery in patients with elevated B7H3 levels (*p* = 0.049). Mucinous adenocarcinoma had high expression of B7H3. Conclusions: Elevated B7H3 expression is associated with reduced oCR rates in LARC, highlighting its potential role as a prognostic biomarker. Further studies with larger cohorts are warranted to validate these findings and explore B7H3-targeted therapies as a treatment strategy for LARC.

## 1. Introduction

Rectal cancer represents approximately a third of colorectal cancers. In 2022, there were 729,000 new cases of rectal cancer and over 340,000 deaths globally [1]. While significant advancements have been achieved both in baseline evaluation and therapy, five-year overall survival for locally advanced rectal cancer is 74% with significant treatment-related morbidity [2].

Locally advanced rectal cancer (LARC) is broadly defined as T3/T4 and/or node-positive disease; however, subgroups can be identified that might benefit from better treatment selection, sequencing, or even the omission of certain interventions [3]. The standard treatment for LARC included neoadjuvant chemoradiation (nCRT) or short-course radiation followed by total mesorectal excision (TME) surgery and mostly adjuvant chemotherapy [4,5]. While this approach obtained favorable local control rates, distant relapses were still significant and reduced overall survival, in part due to poor adherence to adjuvant chemotherapy [6]. Hence, total neoadjuvant treatment (TNT) was introduced to mitigate the risk of distant metastases.

TNT has shown to increase disease-free survival, metastasis-free survival, and rates of pathological (pCR) and clinical complete response (cCR) [7,8,9]. The PRODIGE trial that mandated chemotherapy intensification also proved a benefit in overall survival for TNT [10]. The significant improvement in cCR rates allows patients to undergo non-operative management (NOM), with similar results to those receiving TME, increasing quality of life and decreasing morbidity [11]. Real-world data from the Swedish Colorectal Cancer Registry has shown a threefold increase in overall complete response (oCR) rates (i.e., cCR + pCR), mainly due to the increase in cCR, which seems to partly replace pCR due to patients opting for the watch-and-wait approach [12]. Significant rates of cCR have been obtained with immunotherapy in mismatch repair deficient (dMMR) stage II and III rectal cancer. The landmark phase II trial of Cercek et al. showed that 12/16 patients obtained a durable cCR, with no grade 3 adverse events [13]. A tailored treatment approach is therefore of utmost importance, and alongside validated biomarkers such as clinical TNM, high risk MRI features, or genomic alterations such as microsatellite instability, novel factors can play an important role in patient selection.

**Figure 1:**
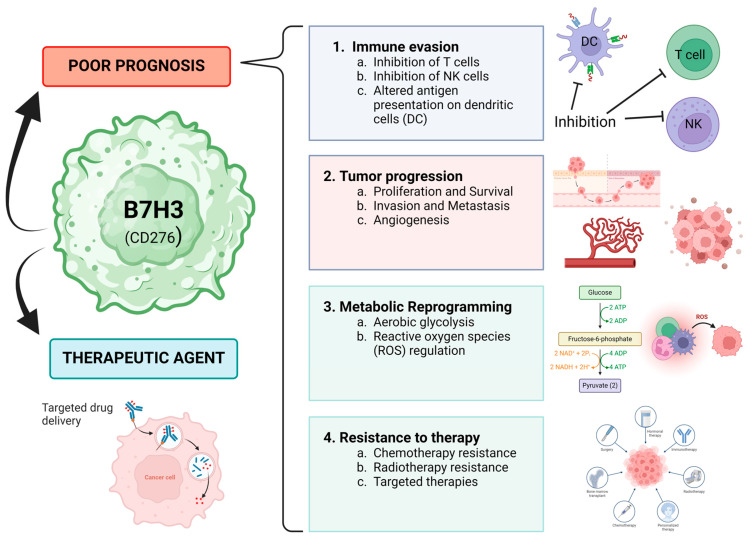
Roles of B7H3 in cancer (NK, natural killer T lymphocytes) [14,17,18,22,23,24].

B7H3, also known as CD276, is an immune checkpoint (IC) protein, a member of the B7 family, which exerts important immune regulation and promotes tumorigenesis, invasiveness, metastasis, and resistance to standard treatment, rendering a consistently poor prognosis [14,15]. B7H3 is mainly expressed in tumor tissues, making it an appealing target for immunotherapy, antibody–drug conjugates (ADCs), CAR-T cells, and radio-immunotherapy, with several clinical trials being active [14]. Figure 1 provides an overview of the primary roles of B7-H3 in cancer.

In colorectal cancer, B7H3 promotes tum or growth and cell viability, modulates the inflammatory response, enhances chemoresistance to oxaliplatin and 5-FU and radio resistance via the B7-H3/KIF15/ERK axis pathway, and inhibits apoptosis [16,17,18,19]. Lu et al. demonstrated that B7H3 expression is associated with inferior disease-free survival (DFS), but not with overall survival (OS) [20]. Ingebrigtsen et al. showed a significant association between the nuclear expression of B7H3 and poor OS, specifically in colon cancer [21].

Considering the increasing role of oCR rates, we aimed to evaluate the expression of B7H3 in locally advanced rectal cancer and its impact on oCR.

## 2. Materials and Methods

### 2.1. Patient Population

This retrospective study was conducted at “Prof. Dr. Ion Chiricuta” Institute of Oncology, Cluj-Napoca, Romania. From the institutional database, we identified 60 consecutive patients diagnosed with AJCC 8th ed. stage I (who declined upfront surgery), II, and III rectal cancers. We divided the cohort into two groups: a validation group and a study group. The rationale for including a validation cohort was to have a chemotherapy- and radiotherapy-naïve group, to serve as a benchmark for our study population considering the local changes induced by chemotherapy and radiotherapy. We considered this necessary as IHC testing was performed on surgical specimens that received prior radiation and/or chemotherapy. The validation group had 11 patients who underwent surgery upfront. The study group contained the remaining 49 patients. Only patients who received long-course nCRT to a minimum of 50Gy in 25 fractions, over the course of five to six weeks, with concurrent Capecitabine 825 mg/m^2^ BID or weekly Fu-Fol (Folinic acid 30 mg/m^2^ and 5FU 600 mg/m^2^) were included. We included patients who received standard nCRT followed by TME surgery and adjuvant chemotherapy, as well as patients who underwent nCRT, neoadjuvant chemotherapy, and surgery as part of TNT. nCRT included doublet chemotherapy, either CAPOX (capecitabine and oxaliplatin) or FOLFOX (folinic acid, 5FU and oxaliplatin). Per institutional protocol, following nCRT, patients with residual disease underwent TME surgery. Patients with cCR were offered the watch-and-wait approach. IHC analysis was performed on the surgical specimens. For patients who achieved a complete response, either clinical or pathological, IHC was performed on baseline, diagnostic biopsy specimens and we documented the clinicopathological data. The study was conducted in accordance with the Declaration of Helsinki and approved by the Institutional Review Board of “Prof. Dr. Ion Chiricuta” Institute of Oncology Cluj-Napoca, Romania (Protocol 303/06.08.2024).

### 2.2. IHC Staining and Evaluation

Hematoxylin–eosin-stained tissue slides were reviewed by an experienced pathologist, who selected representative paraffin-embedded blocks for immunohistochemical (IHC) staining. Slides with 3 μm sections were prepared using an automated immunostainer (BenchMark ULTRA, Ventana Medical Systems, Inc., Tucson, AZ, USA). The antigenic unmasking was based on HIER using CC1 (heating time 30 min. at 95 °C). The primary antibody used was a rabbit monoclonal B7-H3 antibody (Anti-CD276 antibody #EPR20115, 1:100, Abcam, Cambridge, UK). The incubation time was 20 min with the primary antibody and the detection system was OptiView (Roche Diagnostics Interntional AG, Rotkreuz, Switzerland). Using these protocol settings, all cases were optimally stained.

An independent pathologist, blinded to the clinicopathological data, evaluated the immunostained slides. Only tumor cells and tumor stroma were assessed. B7-H3 expression, accounting for tumor heterogeneity, was assessed by recording the mean expression level. Membrane and cytoplasmic staining of tumor cells was considered a single variable, scored for intensity (0 for absent, 1 for weak, 2 for moderate, and 3 for strong) and graded by the percentage of stained cells: 0 for 0–5%, 1 for 6–25%, 2 for 26–50%, and 3 for >50%, as recommended by recent literature [25]. A composite score was calculated by multiplying the percentage grade by the intensity score, with scores <4 considered low expression and scores ≥4 considered high expression. The cut-off was selected based on a study by Li et al. [25]. The rationale for choosing the composite score and this cut-off is that we used whole tissue evaluation (not tissue micro-array cores), and we considered that a two-parameter score would better characterize the expression levels and account for heterogeneity. By using the composite score, if B7H3 was expressed on less than 50% of cells, at least a moderate staining intensity would be needed to consider the tumor positive. Additionally, nuclear and stromal B7-H3 expressions were evaluated using the same intensity grading system (0 for absent, 1 for weak, 2 for moderate, and 3 for strong).

### 2.3. Statistical Analysis

All statistical analyses were conducted using IBM SPSS Statistics v.26.0.0. Distribution analysis was carried out using the Kolmogorov–Smirnov and Shapiro–Wilk tests, supplemented by distribution charts. The independence of variables was assessed using the Chi-square test or Fisher’s exact test, with Yates’ correction for continuity applied when necessary. Dependent groups were compared using paired-samples *t*-tests, while independent groups were analyzed using independent-samples *t*-tests or the Mann–Whitney U test. Linear relationships were tested using Spearman’s correlations. Statistical significance was determined at a threshold of *p* < 0.05 for Pearson’s correlation.

### 2.4. Endpoints

The primary endpoints were the degree of B7H3 expression on tumor cells and its association with oCR and clinicopathological features.

## 3. Results

### 3.1. Study Cohort

The baseline characteristics for the study cohort are listed in Table 1. The study cohort included 49 patients, with a predominance of males (67.3%). Stage III was most frequently observed in our cohort and over half of our patients had low rectal disease. No statistically significant differences were observed between the low- and high-expression groups; however, it is noteworthy that all cases with mucinous histology had high B7H3 expression (*p* = 0.070). 

**Figure 2:**
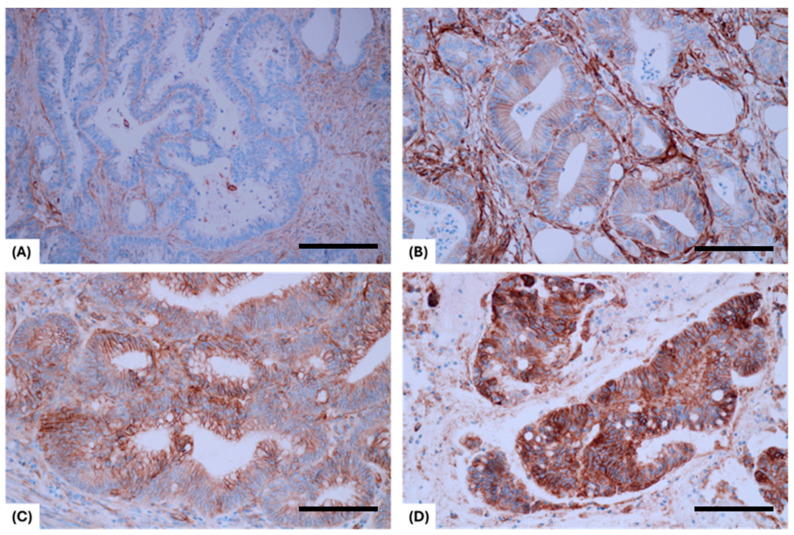
Representative expression patterns for B7H3 expression in rectal tumors. (**A**) No cytoplasmic/membrane staining on diagnostic biopsy tissue in a patient who achieved a pCR (scale bar 100 um at 200×). (**B**) Low cytoplasmic/membrane staining in a patient who received upfront surgery (validation cohort; scale bar 50 um at 400×). (**C**) Moderate cytoplasmic/membrane staining on a surgical specimen, which underwent nCRT (test cohort; scale bar 50 um at 400×). (**D**) High cytoplasmic/membrane staining on a patient with mucinous adenocarcinoma of the rectum, who underwent upfront surgery (validation cohort; scale bar 50 um at 400×).

In our study cohort, B7H3 exhibited mainly cytoplasmic/membrane staining. No nuclear expression was identified. Tumor stroma expression was present in all cases, with most patients having a moderate expression. Figure 2 shows representative B7H3 expression patterns in our study cohort.

**Figure 3:**
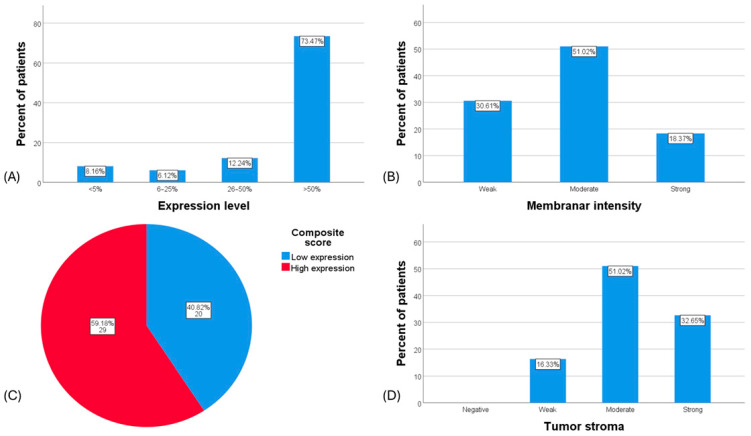
B7H3 expression on cytoplasm/membrane and tumor stroma. (**A**) Percentage of positive stained tumor cells. (**B**) Distribution of stained area intensity. (**C**) Distribution of composite score; low expression = composite score <4, high expression = composite score ≥4, composite score = expression level × membranal intensity. (**D**) Distribution of tumor stroma staining.

Among the 49 patients evaluated, 73.5% (36/49) exhibited B7H3 expression in more than 50% of their tumor cells, whereas only 8.2% (4/49) showed no detectable expression. The intensity of membrane staining was moderate in 51% of cases. According to the composite scoring system, nearly 60% of the patients demonstrated high B7H3 expression, with the remainder exhibiting low expression levels. Figure 3 shows the detailed expression patterns.

**Figure 4:**
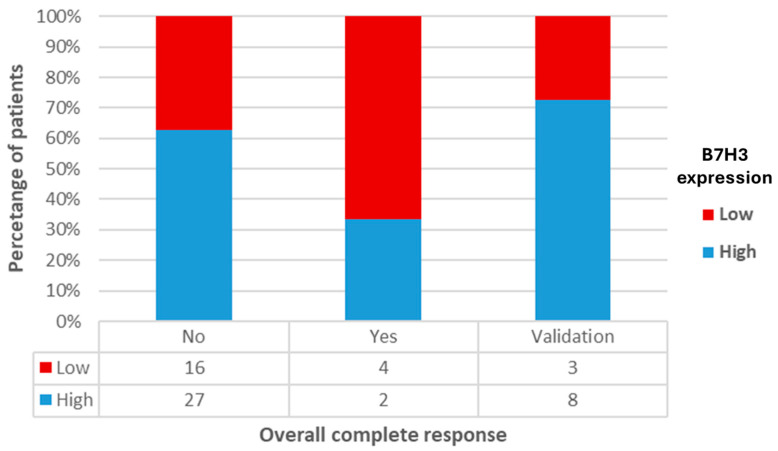
B7H3 expression in overall responders, incomplete responders, and validation cohort.

**Figure 5:**
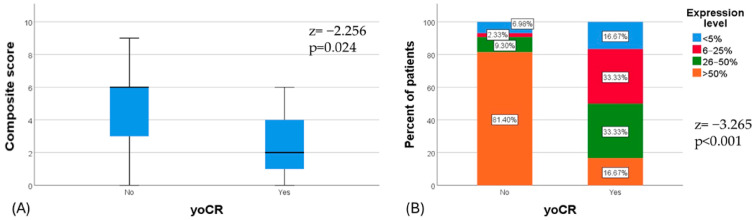
(**A**) Median composite score is significantly lower in patients with oCR compared to patients with residual disease. (**B**) Distribution of percentage positive cells according to oCR.

An oCR to neoadjuvant therapy was observed in 12% (6/49) of patients, with four achieving a pCR and two a cCR. Patients who achieved an oCR exhibited significantly lower B7H3 expression, both in terms of percentage cell staining and composite score, compared to those with residual disease following neoadjuvant treatment (z = −3.265, *p* < 0.001 and z = −2.256, *p* = 0.024, respectively). Figure 4 illustrates the B7H3 expression in overall responders, incomplete responders, and the validation cohort. The median composite score for overall responders was 2 (IQR 4), whereas for non-responders, it was 6 (IQR 3) (Figure 5).

No significant association was found between B7H3 expression and pathological outcomes, apart from the type of surgery performed, as detailed in Table 2. Patients with high B7H3 expression were more likely to undergo non-restorative surgery (*p* = 0.049). Additionally, the majority of patients (83%) experienced tumor downstaging following nCRT.

### 3.2. Validation Cohort

The validation cohort comprised 11 patients with stage I-III rectal cancer who underwent primary surgical resection. B7H3 expression was observed in the tumor cells of all patients, with no nuclear localization detected, and B7H3 was uniformly expressed within the stromal compartment. A high level of B7H3 expression, as determined by the composite score, was present in 72.7% of the patients. Notably, all of the mucinous tumors exhibited high B7H3 expression with a maximum composite score. Two patients had a low-lying tumor, whereas the rest had upper rectal tumors. All specimens were resected with negative margins. The patient characteristics are listed in Table 3.

## 4. Discussion

In this study, we showed that patients achieving an oCR following nCRT for locally advanced rectal cancer have a significantly lower B7H3 expression. To our knowledge, this is the first study evaluating the relationship between B7H3 and CR rates in rectal cancer. Pathological and cCR have been associated with increased DFS and OS [11,26]. There is strong evidence that B7H3 is a negative prognostic factor in colorectal cancer, being linked with poor OS, as shown by Fan et al. in a meta-analysis [27] and Ingebrigtsen et al. in a large retrospective cohort [21]. However, in the latter, only nuclear expression was associated with decreased OS and it was not confirmed in subsequent studies [28]. A large study by Lu et al. showed that the cytoplasmic/membrane expression of B7H3 is associated with inferior disease-free survival, but not OS [20]. The same study, alongside others, demonstrated that B7H3 staining is mainly in the cytoplasm/membrane [20,29,30]. Our study aligns with published data, considering that no patient, either from the study cohort or validation cohort, had nuclear expression of B7H3.

A possible explanation for the poorer response rates seen in tumors overexpressing B7H3 could be that B7H3 activates the ERK 1/2 signaling pathway, known to cause radio resistance in colorectal cancer [17,31]. B7H3 is also associated with chemotherapy resistance by upregulating HK2 and increasing aerobic glycolysis in tumor cells [32]. This has been validated by Zhang et al., who showed that the knockdown of B7H3 reversed chemoresistance to 5FU and oxaliplatin, both drugs that play an essential role in LARC management [33]. 

We found no statistically significant association between clinicopathologic data and the expression of B7H3. Our cohorts were homogeneous and contained mainly patients with stage III rectal cancer. Larger international studies confirmed this lack of a relationship between B7H3 expression and clinicopathologic features [20,28]. Most of these studies included both colon and rectal cancers, with the proportion of rectal cancers varying from 28% to 51%. Considering there is increasing evidence that rectal and colon cancer are two distinct pathological entities, dedicated research for rectal cancer is mandated [34].

The present study included both colorectal adenocarcinoma and mucinous adenocarcinoma and showed that all patients with mucinous histology had high B7H3 expression. This was not statistically significant (*p* = 0.07) due to the small sample size, but it might offer valuable insight into this subgroup of rectal cancer, which has a poor prognosis [35].

Mucinous adenocarcinoma usually has a poorer response to nCRT with less downstaging and more positive circumferential resection margins following TME [36,37,38]. It also harbors more KRAS mutations, which have been shown to preclude achieving a pCR to 5-FU-based chemoradiation [39]. However, adjuvant chemotherapy has been shown to improve OS in mucinous rectal adenocarcinoma [40]. Hence, implementing newer approaches such as TNT or experimental treatments such as antibody–drug conjugates might mitigate the poor outcomes of this subtype.

The analysis in this study was performed on surgical specimens, following nCRT. Radiotherapy has been shown to alter the tumor microenvironment, leading to the upregulation of genes associated with a suppressed immunological profile. This immunosuppressive state is characterized by the upregulation of inflammatory cytokines and immune checkpoint genes (including B7H3), and the accumulation of M2-type macrophages within the tumor [41].

Nonetheless, recent studies have demonstrated that alongside DNA double-strand breaks, radiotherapy also exerts anticancer effects by activating the immune system through the regulation of tumor immunogenicity. This includes enhancing intratumoral immune cell infiltration, enriching neoantigen presence, generating tumor-associated antigen-specific immune cells, and facilitating the activity of major histocompatibility complex class I [42,43,44]. Radiotherapy enriches the immune response in LARC, suggesting that it might facilitate a response to immunotherapy [45]. There are several clinical trials involving B7H3-targeted treatment strategies with early promising results in solid tumor malignancies; hence, priming B7H3 expression through radiotherapy could be an interesting strategy to be further explored [46].

The IHC evaluation methodology utilized in the present study differed slightly from similar studies on colorectal cancer and B7H3. We chose a composite score because we evaluated whole-tissue samples, not tissue micro-array cores. Landmark studies from Ingebrigtsen et al. [28] and Lu et al. [20] both utilized TMAs for IHC and the cut-off between positive and negative expression was staining vs. no staining. Their rationale was that TMA core comprises only a limited part of the tumor, and even staining of a few cells indicates a B7H3-positive tumor. While Lu et al. [20] showed that B7H3 expression was consistent between TMA cores and whole-tissue assessment on randomly selected cases, we did not have the option of building TMA cores and to check for the same consistency. Hence, we chose the composite score, as the methodology used by Li et al. [25] was similar and more suited to our context. Moreover, we considered that a composite score reuniting two parameters would better characterize the specimens and account for heterogeneity.

Our study has several limitations. First, it was a retrospective, mono-institutional study, which might incur selection bias. Second, the study cohort is small, which may reduce the statistical power and generalizability of the findings. Consequently, the findings should be interpreted with caution, and further research with larger cohorts is necessary to validate these results. Moreover, in our study, we did not have paired pre- and post-neoadjuvant therapy samples, which might have better characterized the effect that radiotherapy and chemotherapy have on B7H3 expression. Finally, we did not report survival data due to the insufficient follow-up period since treatment completion.

Future perspectives might include the exploration of PDL1 co-expression or its relationship to microsatellite instability. Preliminary studies have shown that B7H3 is co-expressed with PDL1 in approximately 5% of cases [20] and that it is independently expressed from MSI [47]. This relationship warrants further investigation, particularly in light of evidence from other tumor types, such as breast cancer, where high B7-H3 expression coupled with low PD-L1 levels characterizes “armored-cold” tumors, which exhibit the lowest response rates to various therapies [48].

## 5. Conclusions

High B7H3 expression is associated with reduced oCR rates to neoadjuvant therapy for locally advanced rectal cancer. Further studies with larger cohorts are necessary to validate these results.

## Figures and Tables

**Table 1:** Baseline characteristics of the study cohort.

Variables	Total (*n* = 49)	Low Expression (*n* = 20)	High Expression (*n* = 29)	*p*-Value
Age years, mean (SD)	60.6 (11.2)	60.8 (12.8)	60.5 (10.3)	0.940
Gender				0.126
Male	33 (67.3%)	11 (55%)	22 (75.9%)	
Female	16 (32.7%)	9 (45%)	7 (24.1%)	
ECOG				0.343
0–1	45 (91.8%)	17 (95%)	28 (96.6%)	
2	4 (8.2%)	3 (5%)	1 (3.4%)	
Clinical T stage				0.735
T1	1 (2%)	0 (0%)	1 (3.4%)	
T2	4 (8.2%)	1 (5%)	3 (10.4%)	
T3	36 (73.5%)	16 (80%)	20 (69%)	
T4a	7 (14.3%)	3 (15%)	4 (13.8%)	
T4b	1 (2%)	0 (0%)	1 (3.4%)	
Clinical N stage				0.902
N0	7 (14.3%)	3 (15%)	4 (13.8%	
N1a	2 (4.1%)	1 (5%)	1 (3.4%)	
N1b	12 (24.5%)	5 (25%)	7 (24.2%)	
N2a	5 (10.2%)	1 (5%)	4 (13.8%)	
N2b	23 (46.9%)	10 (50%)	13 (44.8%)	
AJCC Stage				0.638
I	2 (4.1%)	1 (5%)	1 (3.4%)	
II	5 (10.2%)	2 (10%)	3 (10.3%)	
III	42 (85.7%)	17 (85%)	25 (86.3%)	
Tumor location				0.417
Lower rectum	28 (57.1%)	10 (50%)	18 (62.1%)	
Middle rectum	17 (34.7%)	9 (45%)	8 (27.6%)	
Upper rectum	4 (8.2%)	1 (5%)	3 (10.3%)	
MRF ^a^				0.292
MRF positive	18 (38.3%)	9 (47.4%)	9 (32.1%)	
MRF negative	29 (61.7%)	10 (52.6%)	19 (67.9%)	
EMVI ^a^				0.337
EMVI positive	16 (34%)	8 (42.1%)	8 (28.6%)	
EMVI negative	31 (66%)	11 (57.9%)	20 (71.4%)	
TD ^a^				0.204
TD positive	6 (12.8%)	4 (21.1%)	2 (7.1%)	
TD negative	41 (87.2%)	15 (78.9%)	26 (92.9%)	
LPN ^a^				0.452
LPN positive	9 (19%)	5 (26.3%)	4 (14.3%)	
LPN negative	38 (81%)	14 (73.7%)	24 (85.7%)	
CEA at diagnosis (ng/mL) (SD)	7.2 (6.1)	7.6 (7)	7 (5.5)	0.785
Histology				0.070
Adenocarcinoma—NOS	44 (89.8%)	20 (100%)	24 (82.8%)	
Adenocarcinoma—mucinous	5 (10.2%)	0 (0%)	5 (17.2%)	
Tumor grade				0.496
G1	16 (32.7%)	5 (25%)	11 (37.9%)	
G2	26 (53%)	11 (22%)	15 (51.7%)	
G3	7 (14.3%)	4 (20%)	3 (10.4%)	
Distance from anal verge (mm) (SD)	65 (32)	68 (27)	63 (35)	0.631
Neoadjuvant chemotherapy				0.161
Yes	38 (77.5%)	18 (90%)	20 (69%)	
No	11 (22.5%)	2 (10%)	9 (31%)	
Cycles of neoadjuvant chemotherapy, mean (SD)	3.3 (2.9)	3.5 (2.9)	3.2 (3.4)	0.698
Radiotherapy–surgery interval Weeks, mean (SD)	14.1 (7.9)	12.3 (5)	15.2 (9)	0.186

Abbreviations: ECOG, Eastern Cooperative Oncology Group performance status; AJCC, American Joint Committee on Cancer; MRF, mesorectal fascia; EMVI, extra-mural venous invasion; TD, tumor deposits; LPN, lateral pelvic nodes; CEA, carcinoembryonic antigen; NOS, not otherwise specified; ^a^ Only patients who had available baseline MRI, *n* = 47.

**Table 2:** Surgical and pathological results.

Variables	Total (*n* = 49)	Low Expression (*n* = 20)	High Expression (*n* = 29)	*p*-Value
**Procedure**				0.049
Rectosigmoid resection	11 (22.4%)	2 (10%)	9 (31%)	
Anterior resection	11 (22.4%)	7 (35%)	4 (13.8%)	
Abdominoperineal resection	25 (51%)	9 (45%)	16 (55.2%)	
No surgery	2 (4.1%)	2 (10%)	0 (0%)	
Post-operative morbidity a				0.325
Yes	9 (19.1%)	3 (16.7%)	6 (20.7%)	
No	38 (80.9%)	15 (83.3%)	23 (79.3%)	
ypT stage ^a^				0.543
T0	4 (8.6%)	2 (11.1%)	2 (6.9%)	
T1	1 (2.1%)	0 (0%)	1 (3.4%)	
T2	14 (29.8%)	4 (22.2%)	10 (34.5%)	
T3	27 (57.4%)	11 (61.1%)	16 (55.2%)	
T4a	1 (2.1%)	1 (5.6%)	0 (0%)	
ypN stage ^a^				0.279
N0	31 (66%)	12 (66.7%)	19 (65.6%)	
N1a	5 (10.6%)	1 (5.6%)	4 (13.8%)	
N1b	3 (6.4%)	0 (0%)	3 (10.4%)	
N2a	4 (8.5%)	3 (16.7%)	1 (3.4%)	
N2b	4 (8.5%)	2 (11%)	2 (6.8%)	
Resection margins ^a^				0.383
R1	1 (2.1%)	1 (5.6%)	0 (0%)	
R0	46 (97.9%)	17 (94.4%)	29 (100%)	
Modified Ryan TRG ^a^				0.743
0–1	10 (21.3%)	3 (16.7%)	7 (24.2%)	
2	23 (49%)	10 (55.5%)	13 (44.8%)	
3	14 (29.7%)	5 (27.8%)	9 (31%)	
Tumor downstage ^a^				
pT				0.529
Yes	21 (44.7%)	7 (38.9%)	14 (48.3%)	
No	26 (55.3%)	11 (61.1%)	15 (51.7%)	
pN				0.334
Yes	35 (74.5%)	12 (66.7%)	23 (79.3%)	
No	12 (25.5%)	6 (33.3%)	6 (20.7%)	
pT or N				0.455
Yes	39 (83%)	14 (77.8%)	25 (86.2%)	
No	8 (17%)	4 (22.2%)	4 (13.8%)	

Abbreviation: TRG, tumor regression grade. ^a^ Note: *n* = 47 (patients who underwent surgery).

**Table 3:** Baseline characteristics of the validation cohort.

Variable	Number	%
Age (years) (SD)	63 (8.2)	N/A
Gender		
Male	7	63.6
Female	4	36.4
ECOG		
0–1	10	89.9
2	1	10.1
Stage		
I	4	36.3
II	2	18.2
III	5	45.5
Histology		
Adenocarcinoma—NOS	8	72.7
Adenocarcinoma—mucinous	3	27.3
Cytoplasm/Membrane staining		
26–49%	1	9.1
>50%	10	90.9
Membranal intensity		
Weak	3	27.3
Moderate	5	45.4
Strong	5	27.3
Composite score		
Low expression	3	27.3
High expression	8	72.7
Tumor stroma		
Weak	1	9
Moderate	5	45.5
Strong	5	45.5
pT stage		
1	1	9%
2	3	27%
3	7	64%
pN stage		
0	6	54.5%
1a	3	27.3%
1b	1	9.1%
2a	0	0%
2b	1	9.1%

Abbreviation: NOS, not otherwise specified.

## Data Availability

The data presented in this study are available on request from the corresponding author. The data are not publicly available due to patient privacy.
